# Comparative Evaluation of Serum Hepcidin and Fecal Calprotectin for Disease Activity Assessment in Inflammatory Bowel Disease

**DOI:** 10.5152/tjg.2026.25774

**Published:** 2026-05-05

**Authors:** Pırıl Akıncıoğlu, Ülkü Dağlı, Ebru Koca

**Affiliations:** 1 Gastroenterology Clinic, Kepez State Hospital, Antalya, Türkiye; 2 Department of Gastroenterology, Başkent University Faculty of Medicine, Ankara, Türkiye; 3 Department of Hematology, Başkent University Faculty of Medicine, Ankara, Türkiye

**Keywords:** Anemia, Crohn’s disease, fecal calprotectin, hepcidin, inflammatory bowel disease, ulcerative colitis

## Abstract

**Background/Aims:**

Inflammatory bowel disease (IBD), including Crohn’s disease and ulcerative colitis, is a chronic inflammatory disorder commonly associated with iron deficiency anemia and anemia of chronic disease. Hepcidin is a key regulator of iron homeostasis, increasing in response to inflammation and decreasing in iron deficiency. This study evaluated the relationship between serum hepcidin and fecal calprotectin levels and examined the potential role of hepcidin in assessing disease activity and differentiating anemia subtypes.

**Materials and Methods:**

The study included 29 patients with IBD with active disease, 45 in remission, and 34 controls; all participants were evaluated at Başkent University Ankara Hospital in 2014. Complete blood count, serum hepcidin, ferritin, serum iron, total iron-binding capacity (TIBC), C-reactive protein (CRP), and erythrocyte sedimentation rate (ESR) were analyzed in all participants. Fecal calprotectin was additionally measured in patients.

**Results:**

Hepcidin levels were significantly higher in patients with both active disease and in remission compared with controls (*P* < .05). Hepcidin levels were negatively correlated with hemoglobin, hematocrit, and ferritin and positive correlated with TIBC and ESR (*P *< .05). Fecal calprotectin levels were significantly higher in patients with active disease and were positively correlated with CRP and clinical activity scores (*P *< .01). However, no significant correlation was found between hepcidin and fecal calprotectin (*P *> .025).

**Conclusion:**

Fecal calprotectin was strongly associated with disease activity, whereas hepcidin was not, suggesting that hepcidin may have limited utility for disease activity assessment. Hepcidin may primarily reflect systemic iron metabolism and anemia of inflammation. Further studies are needed to clarify its clinical role.

**Main Points**

- Fecal calprotectin is the most reliable marker of mucosal inflammation in inflammatory bowel disease (IBD).
- Hepcidin reflects systemic inflammation and iron dysregulation and may assist in the assessment of anemia but is not suitable for monitoring mucosal disease activity.
- A dual-biomarker approach (calprotectin + hepcidin) provides complementary insights into IBD pathophysiology.

## Introduction

Inflammatory bowel disease (IBD), including Crohn’s disease and ulcerative colitis, is characterized by chronic gastrointestinal inflammation. Anemia is a common extraintestinal complication of IBD, affecting a significant proportion of patients.^1^ The pathophysiology of anemia in IBD is multifactorial, involving iron deficiency, anemia of chronic disease, and, less commonly, other forms such as hematinic deficiency anemia. Hepcidin, a hepatic peptide hormone that regulates iron homeostasis, plays a central role in the pathogenesis of anemia of chronic disease. Hepcidin levels increase during inflammatory states, leading to reduced iron absorption and mobilization.^2^ In IBD, this dysregulation poses significant challenges in the diagnosis and management of anemia, highlighting the need for reliable biomarkers to distinguish underlying etiologies. The importance of this issue is further heightened in the country, where increasing incidence and earlier age of onset contribute to a growing public health burden.^3^

Calprotectin is a protein released by activated neutrophils and serves as an essential biomarker of intestinal inflammation in IBD. Fecal calprotectin levels correlate strongly with disease activity and mucosal inflammation and serve as a valuable noninvasive tool for diagnosis, disease monitoring, and therapeutic follow-up.^4^^-^^6^ Elevated fecal calprotectin reflects neutrophil migration to the intestinal mucosa and serves as a surrogate marker of mucosal inflammation and disease severity.

Recent evidence suggests an association between serum hepcidin and fecal calprotectin levels in IBD, indicating that hepcidin may not only serve as a regulator of iron metabolism but also reflect systemic inflammatory activity.^7^ Investigating the correlation between these 2 biomarkers may provide insight into the interaction between inflammation and iron regulation, elucidating the complex relationship underlying anemia in IBD.

This study aims to assess the correlation between serum hepcidin and fecal calprotectin levels in patients with IBD and to investigate the potential role of hepcidin in disease activity assessment and differentiation of anemia etiologies.^2^^,^^8^ By establishing this relationship, this study aims to improve the understanding of anemia mechanisms in IBD and improve clinical decision-making in anemia management in this patient population.

## Materials and Methods

### Study Design and Ethical Approval

This prospective study included patients aged 18-65 years with a diagnosis of IBD who were followed up at the Gastroenterology Outpatient Clinic of Başkent University Ankara Hospital. The study protocol was reviewed and approved by the Ethics Committee of Başkent University School of Medicine (Date: January 24, 2014, No: 14/12). All procedures were conducted in accordance with the ethical standards of the Declaration of Helsinki. Written informed consent was obtained from all participants before enrollment. Although data were originally collected in 2014, this study re-evaluated fecal calprotectin levels using currently accepted disease activity thresholds reported in contemporary literature (i.e., >150 µg/g for active disease and >250 µg/g for severe activity), and the analyses and interpretation were updated accordingly. Nevertheless, the findings should be interpreted within the diagnostic and therapeutic context of 2014.

### Patient Selection

Patients were eligible if they had a documented diagnosis of either Crohn’s disease or ulcerative colitis based on standard clinical, radiological, endoscopic, and histopathological criteria. Exclusion criteria included renal or hepatic dysfunction, connective tissue disease, malignancy, active infection, use of nonsteroidal anti-inflammatory drugs, iron replacement therapy, and other conditions that could affect hepcidin or iron parameters, including hemoglobinopathies, chronic inflammatory comorbidities, and sex-specific physiologic states (e.g., pregnancy/lactation or marked menstrual-related blood loss at the time of sampling). The study group consisted of 74 patients with IBD, including 29 with active disease and 45 in remission (Table 1). In the present study, disease activity was primarily assessed using clinical indices. The control group comprised 34 healthy volunteers without systemic illness or relevant medication use and without conditions known to affect hepcidin or iron parameters.

All participants underwent detailed clinical assessment and laboratory testing. Disease activity was determined using the Crohn’s Disease Activity Index for patients with Crohn’s disease and the Truelove–Witts severity index for those with ulcerative colitis. Fecal calprotectin was additionally evaluated using contemporary cutoffs commonly applied in current practice (>150 µg/g to indicate active inflammation and >250 µg/g to indicate more severe activity) to support sensitivity analyses. Endoscopic disease activity scores (e.g., Simple Endoscopic Score for Crohn’s Disease (SES-CD) and Ulcerative Colitis Endoscopic Index of Severity) were available and used in supplementary analyses to assess the relationship between biomarkers and mucosal inflammation. Disease location and extent were also systematically recorded and analyzed according to the Montreal classification.

The following laboratory parameters were obtained: complete blood count, erythrocyte sedimentation rate (ESR), serum iron (SI), total iron-binding capacity (TIBC), ferritin, and C-reactive protein (CRP). Transferrin saturation (TS) was calculated using the formula: TS (%) = (SI/TIBC) × 100.

Anemia was defined as hemoglobin <13 g/dL in men and <12 g/dL in women. Patients with ferritin <30 ng/mL and TS <16% were classified as having iron deficiency anemia, those with ferritin >100 ng/mL as having anemia of chronic disease, and those with intermediate ferritin (30-100 ng/mL) as having mixed anemia.

Hepcidin concentrations were measured using a commercial enzyme-linked immunosorbent assay (ELISA) kit (Uscn Life Science Inc., Cloud-Clone Corp., Katy, TX, USA). Serum samples were stored at −80°C until analysis. The assay sensitivity was <26.4 pg/mL, with intra-assay coefficient of variation (CV) <10% and inter-assay CV <12%. Fecal calprotectin was quantified using the BÜHLMANN Quantum Blue® immunoassay system. A cutoff of 150 μg/g was used to differentiate organic from functional gastrointestinal conditions; values >250 μg/g were considered indicative of active organic disease.

### Statistical Analysis

All data were analyzed using Statistical Package for the Social Sciences (SPSS) for Windows, version 21.0 (IBM SPSS Corp.; Armonk, NY, USA). The Kolmogorov–Smirnov test was used to assess normality, and Levene’s test was used for homogeneity of variances. Descriptive statistics were presented as mean ± SD for normally distributed variables and median (minimum–maximum) for skewed data. Group comparisons were performed using 1-way analysis of variance (ANOVA) when assumptions were met; otherwise the Mann–Whitney *U* or Kruskal–Wallis tests were used. Post hoc analyses were carried out with Tukey’s Honestly Significant Difference (HSD) or Conover’s multiple comparison test to identify the source of significant differences. Categorical variables were analyzed using Pearson’s chi-square test, and correlations between numerical variables were assessed using Spearman’s rank correlation. To account for the age imbalance between controls and patients and the potential confounding effect of age on hepcidin, multivariable models for between-group comparisons of hepcidin were adjusted for age and sex a priori (forced-entry adjustment). Additional covariates were selected based on univariate screening (*P* < .10). Differences in hepcidin levels were further assessed using multiple linear regression, adjusting for potential confounders. For fecal calprotectin, sensitivity analyses were conducted using contemporary activity thresholds (>150 µg/g and >250 µg/g), and between-group comparisons were repeated based on these cutoffs. Logarithmic transformation was applied to non-normally distributed hepcidin levels. Statistical significance was defined as *P* < .05.

## Results

### Study Population and Baseline Characteristics

A total of 74 patients with IBD were included in the study, comprising both Crohn’s disease (n = 24) and ulcerative colitis (n = 50) cases, along with 34 healthy controls. The mean age was 29.5 ± 5.8 years in the controls, 41.9 ± 12.0 years in patients in remission, and 39.9 ± 11.8 years in those with active disease. Hematological indices varied across groups. Sex distribution did not differ significantly among groups (*P* = .616) (Table 2).

### Hematological and Biochemical Findings

Hemoglobin levels were significantly lower in patients with active disease compared with those in remission and controls (*P *< .01). Similarly, hematocrit and mean corpuscular volume (MCV) were lower in the active group, reflecting the impact of chronic inflammation and anemia of mixed etiology. Platelet counts were significantly higher in active disease than in remission and controls (*P *< .05), consistent with an inflammatory response. SI levels were lowest in the active IBD group, whereas ferritin levels were elevated, reflecting its role as an acute-phase reactant. TS was significantly lower in active disease (*P *< .01), whereas TIBC did not differ significantly between groups. Inflammatory markers, including C-reactive protein (CRP) and ESR, were significantly higher in the active group than in remission and controls (*P* < .001). Both markers were positively correlated with clinical activity scores.

### Serum Hepcidin and Fecal Calprotectin Levels and Disease Activity

The primary objective of this study was to evaluate serum hepcidin levels in relation to disease activity. Median hepcidin concentrations were higher in patients with active IBD (969.0 pg/mL) than in those in remission (776.5 pg/mL) and controls (424.8 pg/mL) (overall *P *< .001). Although patients in remission exhibited slightly higher hepcidin levels than controls, this difference was attenuated after adjustment for age (and sex) and should be interpreted with caution given the control group’s younger age. Hepcidin concentrations tend to increase with advancing age; however, this is largely attributed to age-associated changes such as increased iron stores and chronic low-grade inflammation, rather than aging itself.

Importantly, elevations in hepcidin appeared to parallel systemic inflammation and iron-restricted erythropoiesis rather than serving as a robust signal of clinical disease activity. When stratified by disease subtype, patients with ulcerative colitis and Crohn’s disease exhibited similar patterns of hepcidin elevation during active disease phases. No significant differences in absolute hepcidin concentrations were observed between ulcerative colitis and Crohn’s disease within the same disease activity category (*P* > .05). Comparisons of laboratory indices between remission and control groups suggested that subtle abnormalities may persist despite clinical quiescence (e.g., higher ferritin in remission vs. controls), which may reflect residual inflammation or ongoing iron restriction. Spearman correlation analyses demonstrated modest correlations between serum hepcidin and inflammatory/hematologic parameters; however, clinical activity scores did not correlate with hepcidin in remission, active disease, or the overall IBD cohort. In both ulcerative colitis and Crohn’s disease, anemic patients exhibited higher hepcidin levels and lower ferritin levels compared with nonanemic patients. Notably, hepcidin levels remained elevated despite low ferritin concentrations, suggesting inflammation-driven dysregulation of iron homeostasis.

Median fecal calprotectin levels were also significantly higher in active IBD than in remission (*P* < .001). Correlation analyses demonstrated significant positive associations between fecal calprotectin and CRP, ESR, and clinical activity scores across all cases (*P *< .01). However, no significant correlations were observed with other variables, including hepcidin (*P *> .05). When analyzed within activity subgroups, fecal calprotectin showed no significant correlations with CRP, ferritin, hemoglobin, ESR, or hepcidin (*P* > .025). Likewise, clinical activity scores did not correlate with hepcidin in remission (*r* = 0.031, *P* = .838), active disease (*r* = 0.059, *P* = .760), or the combined IBD cohort (*r* = 0.077, *P* = .516). Subgroup analysis by disease type revealed no significant associations between hepcidin and clinical activity indices in Crohn’s disease (*r* = 0.114, *P* = .595) or ulcerative colitis (*r* = 0.092, *P* = .527). Similarly, fecal calprotectin and hepcidin levels showed no significant correlations within Crohn’s (*r* = 0.176, *P* = .410) or ulcerative colitis subgroups (*r* = 0.030, *P* = .835) (Table 3 and Table 4). When fecal calprotectin thresholds of >150 µg/g and >250 µg/g were applied, differences between active and remission groups did not reach statistical significance (*P* = .107 and *P* = .151, respectively). Hepcidin concentrations also did not differ significantly across these fecal calprotectin threshold-based strata (*P* = .492). Likewise, hepcidin concentrations did not differ significantly between Crohn’s disease (881.6 pg/mL) and ulcerative colitis (816.4 pg/mL) (*P* = .995). Patients were categorized according to the Montreal classification as E1 (n = 22, 51.2%), E2 (n = 11, 25.6%), and E3 (n = 10, 23.3%). Given the non-normal distribution observed in most subgroups, hepcidin and fecal calprotectin levels were summarized as median (min–max). Median hepcidin levels were 675 ng/mL (166-2605) in E1, 664 ng/mL (62-1907) in E2, and 906 ng/mL (80-1709) in E3, with no statistically significant difference across groups (Kruskal–Wallis *χ*^2^ = 0.448, *df* = 2, *P* = .799, *ε*^2^ = 0.0107). In contrast, fecal calprotectin levels demonstrated a marked and progressive increase with greater disease extent, with median values of 25.5 µg/g (14-2000) in E1, 268 µg/g (89-2000) in E2, and 1562 µg/g (110-2000) in E3. This difference was statistically significant (Kruskal–Wallis *χ*^2^ = 24.055, *df* = 2, *P *< .001, ε^2^ = 0.5727), reflecting a large effect size and indicating a strong association with disease extent. Fecal calprotectin levels were significantly positively correlated with the Mayo endoscopic score (MAYO) , an endoscopic activity index used in ulcerative colitis (Spearman’s rho *r* = 0.697, *P* < .001), whereas serum hepcidin levels showed no significant association with disease activity (*P* > .05). In patients with Crohn’s disease, additional nonparametric 1-way ANOVA (Kruskal–Wallis) analyses across disease location subgroups revealed no statistically significant differences in biomarker levels for either hepcidin (*χ*^2^ = 4.05, *df* = 6, *P* = .670) or fecal calprotectin (*χ*^2^ = 6.58, *df* = 6, *P* = .361). In patients with Crohn’s disease, neither fecal calprotectin nor serum hepcidin showed a significant association with endoscopic disease activity as assessed by the SES-CD (*P *> .05), and no correlation was observed between these biomarkers. Similarly, nonparametric analyses (Kruskal–Wallis) across disease location subgroups revealed no statistically significant differences in biomarker levels for either hepcidin (*χ*^2^ = 4.05, *df* = 6, *P* = .670) or fecal calprotectin (*χ*^2^ = 6.58, *df* = 6, *P* = .361). Overall, unlike in ulcerative colitis, these findings indicate that neither fecal calprotectin nor hepcidin demonstrated a significant relationship with disease activity or extent in Crohn’s disease within this cohort. These findings suggest that, while fecal calprotectin closely reflects disease extent in ulcerative colitis, neither biomarker appears to be significantly influenced by disease location in Crohn’s disease within this cohort. This observation may, at least in part, be explained by the well-established tendency of fecal calprotectin to be more markedly elevated in colonic involvement compared with isolated ileal disease, which may attenuate detectable differences across heterogeneous disease location subgroups.

Among patients with IBD, 12 (16.2%) had iron deficiency anemia, 3 (4.05%) had anemia of chronic disease, and 1 (1.35%) had combined anemia. Iron deficiency anemia was observed in 7 patients with active disease and 5 in remission, while anemia of chronic disease was seen in 2 patients with active disease and 1 in remission. Only 1 patient in the active group exhibited both types of anemia. Median hepcidin levels were significantly higher in patients with anemia (1187.2 pg/mL) compared with those without anemia (699.1 pg/mL) (*P* = .013). No significant differences in hepcidin levels were observed among the control group, nonanemic remission, and nonanemic active patients (*P* = .093). However, when comparing controls with anemic subgroups, hepcidin levels were significantly elevated in anemic active patients (*P *< .001), whereas differences between anemic remission and active patients, as well as between anemic remission patients and controls, did not reach statistical significance (*P* = .052 and *P* = .240, respectively). These anemia subtype comparisons are underpowered because of the very small sample sizes (e.g., anemia of chronic disease n = 3; combined anemia n = 1); therefore, non-significant findings should be interpreted as no association detected rather than evidence of no true difference.

Multivariable linear regression analysis was conducted to assess independent predictors of hepcidin levels. Age and sex were included a priori in the models, and additional variables were entered based on univariate screening (*P *< .10). After adjustment, active IBD remained associated with higher hepcidin compared with controls, whereas the remission–control contrast was less consistent and should be interpreted with caution because of the age imbalance and residual confounding. Among biochemical variables, lower hemoglobin and indices of iron availability (e.g., TS) were inversely associated with hepcidin in multivariable modeling.

## Discussion

This study investigated the interplay between clinical symptoms, laboratory biomarkers, and inflammatory activity in IBD, with a focus on the roles of fecal calprotectin and hepcidin in disease activation and anemia. Consistent with the existing literature, patients predominantly presented with intestinal symptoms such as bloody stool and abdominal pain. Anemia emerged as the most frequent systemic manifestation, likely resulting from a combination of intestinal blood loss, malabsorption, dietary insufficiency, and medication effects, with iron deficiency anemia being the most common form.^9^^-^^11^ The current study confirmed that iron deficiency anemia remains the predominant anemia subtype in both active and remission phases. Patients with active IBD exhibited significantly lower hemoglobin levels compared with both patients in the remission phase and healthy controls (*P* = .002), whereas remission-phase patients did not differ significantly from controls, corroborating the findings of Alves et al,^12^ who reported no significant between-group disparity across disease types (*P* = .77). Reflecting broader pathophysiology, parameters such as MCV, red cell distribution width (RDW), TS, and TIBC exhibited expected alterations in patients with IBD, with lower MCV, TS, in the patient group, alongside elevated RDW and TIBC. These findings reinforce the role of chronic inflammation and disrupted iron homeostasis in IBD-associated anemia.

Unlike systemic inflammatory markers such as CRP, interleukin-6 (IL-6), or ferritin, fecal calprotectin reflects neutrophil activity within the intestinal mucosa, making it a more specific indicator of local inflammation.^13^ Consistent with this, fecal calprotectin levels were significantly higher in patients with active disease compared with those in remission in both IBD cohorts (*P *< .001), supporting its established role in clinical practice. Given that fecal calprotectin thresholds for disease activity have evolved over time, the cohort was additionally re-analyzed using contemporary cutoffs (>150 µg/g for active disease and >250 µg/g for more severe activity), which did not materially alter the lack of association between hepcidin and fecal calprotectin.

When endoscopic activity was evaluated in Crohn’s disease, neither hepcidin nor fecal calprotectin levels differed significantly across SES-CD activity groups (all *P *> .05). However, while hepcidin levels remained relatively stable, fecal calprotectin showed a numerical increase with higher endoscopic activity, suggesting a closer relationship with mucosal inflammation. Similarly, in ulcerative colitis, fecal calprotectin levels were significantly higher in patients with active disease compared with those in remission (*P *< .001), whereas hepcidin levels did not differ between groups (*P* = .869). These findings are in line with the previous literature indicating that colonic inflammatory activity is more closely reflected by fecal calprotectin than by systemic markers such as hepcidin.^14^

Hepcidin, a liver-derived peptide that regulates iron homeostasis by binding to ferroportin and restricting iron export, is increasingly understood as an acute-phase reactant that is elevated in systemic inflammation.^15^ Prior research has demonstrated that in IBD, serum hepcidin correlates positively with CRP and ferritin and inversely with transferrin and soluble transferrin receptor levels, supporting its role in distinguishing iron deficiency anemia from anemia of chronic disease.^16^ In this cohort, serum hepcidin levels were higher in IBD groups than in controls, but it did not reliably differentiate active from remission states, and clinical activity scores did not correlate with hepcidin in subgroup analyses. A 2024 systematic review by Ferrari et al^8^ reported conflicting hepcidin findings across IBD cohorts and highlighted substantial methodological heterogeneity in hepcidin quantification (e.g., immunoassays such as ELISA vs. mass spectrometry, and whether hepcidin-25 or total hepcidin is measured), which may partly explain inconsistencies between studies and should be considered when comparing results across cohorts. Importantly, detailed data on ongoing IBD therapies (e.g., corticosteroids, immunomodulators, and biologics) were not available for the present analyses; therefore, treatment-related confounding could not be assessed or adjusted for, and causal interpretations should be avoided.

In this cohort, no significant correlation was observed between hepcidin and fecal calprotectin. This divergence may reflect their differing biological scopes: fecal calprotectin primarily reflects intestinal neutrophil-driven inflammation, whereas hepcidin more closely reflects systemic inflammatory signaling and iron regulation. Accordingly, the findings of the current study suggest that hepcidin provides limited incremental value for assessing clinical disease activity when fecal calprotectin and clinical indices are available; however, it may remain informative for characterizing inflammation-related iron dysregulation. Based on the post hoc analysis, the critical correlation coefficient (critical *r*) required for statistical significance was relatively high (*r* ≈ 0.47), indicating that only strong correlations could be detected with the available sample size. Therefore, weaker but potentially clinically relevant associations may not have reached statistical significance.

Limitations of this study include: (i) a modest overall sample size and very small numbers in key subgroups (particularly anemia subtypes), which limit statistical power and increase the risk of false-negative findings; (ii) marked age imbalance between controls and patient groups, which may influence hepcidin and required age-adjusted analyses; (iii) use of a dataset originating from 2014, with subsequent diagnostic/therapeutic advances limiting direct generalizability to contemporary practice; and (iv) exclusion of certain factors known to affect hepcidin regulation, particularly obesity. Obesity is characterized by chronic low-grade inflammation and increased cytokine activity, especially IL-6, which can stimulate hepcidin production independently of intestinal disease activity.

Within these limitations, the primary message of this study is that fecal calprotectin robustly tracks clinical disease activity, whereas serum hepcidin does not correlate with clinical activity indices or fecal calprotectin in this cohort. Thus, the data do not support a dual-biomarker disease activity assessment strategy based on hepcidin in addition to fecal calprotectin; rather, hepcidin may be more relevant as a systemic marker of iron metabolism and inflammation-related anemia.

Future studies should use larger, longitudinal cohorts with contemporaneous endoscopic scoring and careful characterization of disease location/extent while adjusting for age, sex, body mass index, and treatment exposures. Standardization of hepcidin measurement (assay type and analyte definition) will also be essential. Such designs could clarify whether hepcidin has a role in specific phenotypes (e.g., anemia of chronic disease) or in predicting iron-restricted erythropoiesis over time, rather than in routine disease activity monitoring.

In conclusion, fecal calprotectin remains a sensitive indicator of intestinal inflammatory activity in IBD. In this cohort, serum hepcidin did not correlate with clinical disease activity indices or fecal calprotectin, suggesting limited utility for disease activity assessment. Hepcidin may instead be interpreted as a systemic marker of inflammation-driven alterations in iron metabolism and anemia. Larger contemporary studies with endoscopic correlation and standardized hepcidin assays are needed before its clinical integration into routine IBD monitoring can be recommended.

## Figures and Tables

**Table 1.** Distribution of Disease Activity According to Inflammatory Bowel Disease Subtype

**Disease Activity**	**Ulcerative Colitis** **(n = 50)**	**Crohn’s Disease** **(n = 24)**	**Total** **(N = 74)**	P*
Remission	27 (54.0)	18 (75.0)	45 (60.8)	
Active	23 (46.0)	6 (25.0)	29 (39.2)	**.083**
Total	50 (100)	24 (100)	74 (100)	

Data are presented as n (%). *P* value is calculated using the chi-square test (*χ*^2^= 3.00,* df =* 1, N = 74). Disease activity was defined using clinical indices (Crohn’s Disease Activity Index for Crohn’s disease and Truelove–Witts criteria for ulcerative colitis).

**Table 2.** Demographic and Baseline Clinical Characteristics of the Study Population Stratified by Disease Activity Groups

**Variables**	**Control (n = 34)**	**Remission (n = 45)**	**Active (n = 29)**	*P*
Age	29.5 ± 5.8^a,b^	41.9 ± 12.0^a^	39.9 ± 11.8^b^	**<.001**
Sex				.616
Male, n (%) Female, n (%)	14 (41.2) 20 (58.8)	23 (51.1) 22 (48.9)	15 (51.7) 14 (48.3)	
Hb	14.3 ± 1.39^x^	13.8 ± 1.53	13.0 ± 1.75^x^	**.003**
HCT	43.4 ± 3.8^x^	42.6 ± 4.1^y^	40.1 ± 4.7^x,y^	**.007**
WBC	6.7 (3.2-11.9)^x,z^	7.9 (4.4-17.5)^y,z^	9.0 (6.4-15.6)^x,y^	**<.001**
PLT	246.5 (166.0-488.0)^x,z^	278.0 (168.0-528.0)^y,z^	352.0 (204.0-560.0)^x,y^	**<.001**
MCV	88.2 (78.4-95.2)^x,z^	84.6 (59.5-92.1)^z^	83.3 (67.8-93.0)^x^	**<.001**
RDW	12.1 (10.7-16.1)^x,z^	13.4 (11.2-23.6)^z^	13.3 (10.7-25.8)^x^	**.009**
Ferritin	47.2 (6.5-289.0)	33.5 (1.9-269.0)	22.4 (2.7-359.0)	.093
SI	97.5 (44.0-182.0)^x,z^	60.0 (15.0-157.0)^z^	48.0 (11.0-120.0)^x^	**<.001**
TIBC	223.0 ± 60.0^x,z^	275.8 ± 68.0^z^	272.6 ± 64.4^x^	**<.001**
TS	47.0 (13.0-105.0)^x,z^	21.0 (4.0-84.0)^z^	15.0 (3.0-57.0)^x^	**<.001**
CRP	1.06 (0.20-5.70)^x^	1.40 (0.20-90.32)^y^	4.67 (0.42-255.65)^x,y^	**<.001**
ESR	2.0 (2.0-14.0)^x,z^	7.0 (2.0-42.0)^y,z^	19.0 (7.0-189.0)^x,y^	**<.001**
Hepcidin	424.8 (11.8-1591.2)^x,z^	776.5 (51.7-2605.0)^z^	969.0 (80.0-3705.9)^x^	**.017**

Values are presented as mean ± SD or median (range).

^a^Statistically significant difference between the control and remission groups (*P* < .001).

^b^Statistically significant difference between the control and active disease groups (*P* < .001).

^x^Significant difference between control and active groups (*P *< .05).

^y^Significant difference between remission and active groups (*P *< .05).

^z^Significant difference between control and remission groups (*P* < .05).

CRP, C-reactive protein; ESR, erythrocyte sedimentation rate; Hb, hemoglobin; HCT, hematocrit; MCV, mean corpuscular volume; PLT, platelet; RDW, red cell distribution width; SI, serum iron; TIBC, total iron-binding capacity; TS, transferrin saturation; WBC, white blood cell.

**Table 3.** Correlation of Hepcidin with Age and Laboratory Parameters Across Study Groups

	**All Patients**		**Control**		**Remission**		**Active **	
	**Correlation Coefficient, *r***	*P* **^a^**	**Correlation Coefficient, *r***	*P* **^b^**	**Correlation Coefficient, *r***	*P* **^b^**	**Correlation Coefficient, *r***	*P* **^b^**
Age	0.087	.369	0.035	.843	−0.116	.449	−0.051	.794
Hb	−0.218	**.024**	−0.285	.103	0.049	.748	−0.413	.026
HCT	−0.209	**.030**	−0.250	.154	0.041	.789	−0.430	.020
WBC	0.162	.094	0.053	.766	0.009	.951	−0.038	.843
PLT	0.184	.057	0.108	.545	0.222	.142	−0.070	.720
MCV	−0.200	**.038**	0.339	.050	−0.218	.150	−0.395	.034
RDW	0.134	.166	−0.113	.526	−0.117	.443	0.513	**.004**
Ferritin	−0.268	**.005**	−0.243	.167	−0.149	.329	−0.382	.041
SI	−0.178	.066	0.031	.860	0.109	.478	−0.410	.027
TIBC	0.295	**.002**	0.129	.467	0.179	.239	0.262	.170
TS	−0.245	**.010**	0.041	.817	0.013	.933	−0.455	**.013**
CRP	−0.060	.539	−0.305	.079	−0.116	.449	−0.205	.286
ESR	0.208	**.031**	0.273	.119	−0.075	.624	0.121	.532

CRP, C-reactive protein; ESR, erythrocyte sedimentation rate; Hb, hemoglobin; HCT, hematocrit; MCV, mean corpuscular volume; PLT, platelet; RDW, red cell distribution width; SI, serum iron; TIBC, total iron-binding capacity; TS, transferrin saturation; WBC, white blood cell.

**Table 4.** Correlation Coefficients and Significance Levels Between Fecal Calprotectin and Other Clinical Parameters in the Patient Cohort

	**All Patients**		**Remission**		**Active**	
	**Correlation Coefficient, *r***	*P* **^a^**	**Correlation Coefficient, *r***	*P* **^b^**	**Correlation Coefficient, *r***	*P* **^b^**
CRP	0.391	**<.001**	0.127	.216	−0.045	.816
Ferritin	−0.149	.204	−0.193	.204	0.183	.342
Hb	−0.249	**.033**	−0.138	.364	0.084	.666
ESR	0.532	**<.001**	0.350	.019	0.040	.836
Activity score	0.592	**.004**	0.321	.226	−0.441	.381
Hepcidin	0.086	.464	0.099	.518	−0.149	.439

^a^*P* < .05 was considered statistically significant.

^b^Bonferroni-adjusted significance level: *P* < .025

CRP, C-reactive protein; ESR, erythrocyte sedimentation rate; Hb, hemoglobin.

## Data Availability

The data that support the findings of this study are available on request from the corresponding author.

## References

[1] Kosmala W Negishi T Thavendiranathan P Incremental value of myocardial work over global longitudinal strain in the surveillance for cancer-treatment-related cardiac dysfunction: a case-control study J Clin Med. 2022;11(4):912. (doi: 10.3390/jcm11040912) PMC8880507 35207185

[2] Acar Ş Akyıldız M Karaali Z The evaluation of the role of (pro)hepcidin in anemia encountered in inflammatory bowel diseases. J Clin Pract Res. 2023;45(3):222 226. (doi: 10.14744/etd.2022.95881) 41255814 PMC12478653

[3] Durak MB Cagin YF Balkan A Three-decade analysis of inflammatory bowel disease in Turkiye: a multicenter study (1993-2024). Turk J Gastroenterol. 2025;36(12):822 833. (doi: 10.5152/tjg.2025.25063) . 41340405 PMC12684269

[4] Erbayrak M Türkay C Eraslan E The role of fecal calprotectin in investigating inflammatory bowel diseases. Clinics (Sao Paulo). 2009;64(5):421 425. (doi: 10.1590/S1807-59322009000500009) 19488608 PMC2694246

[5] Wang W Cao W Zhang S The role of calprotectin in the diagnosis and treatment of inflammatory bowel disease. Int J Mol Sci. 2025;26(5):1996. (doi: 10.3390/ijms26051996) PMC11900593 40076618

[6] Hu T Zhang Z Song F Evaluation of mucosal healing in ulcerative colitis by fecal calprotectin vs. fecal immunochemical test: a systematic review and meta-analysis. Turk J Gastroenterol. 2023;34(9):892 901. (doi: 10.5152/tjg.2023.22812) 37427885 PMC10544655

[7] Paköz ZB Çekiç C Arabul M An Evaluation of the correlation between hepcidin serum levels and disease activity in inflammatory bowel disease. Gastroenterol Res Pract. 2015;2015:810942. (doi: 10.1155/2015/810942) PMC4299302 25628652

[8] Ferrari F Carini M Zanella I Potential diagnostic role of hepcidin in anemic patients affected by inflammatory bowel disease: a systematic review. Diagnostics (Basel). 2024;14(4):375. (doi: 10.3390/diagnostics14040375) PMC10887700 38396414

[9] Kaitha S Bashir M Ali T. Iron deficiency anemia in inflammatory bowel disease. World J Gastrointest Pathophysiol. 2015;6(3):62 72. (doi: 10.4291/wjgp.v6.i3.62) 26301120 PMC4540708

[10] Akpınar H Çetiner M Keshav S Diagnosis and treatment of iron deficiency anemia in patients with inflammatory bowel disease and gastrointestinal bleeding: iron deficiency anemia working group consensus report. Turk J Gastroenterol. 2017;28(2):81 87. (doi: 10.5152/tjg.2017.17593) 28119272

[11] Filmann N Rey J Schneeweiss S Prevalence of anemia in inflammatory bowel diseases in European countries: a systematic review and individual patient data meta-analysis. Inflamm Bowel Dis. 2014;20(5):936 945. (doi: 10.1097/01.MIB.0000442728.74340.fd) 24572205

[12] Alves RA Miszputen SJ Figueiredo MS. Anemia in inflammatory bowel disease: prevalence, differential diagnosis and association with clinical and laboratory variables. Sao Paulo Med J. 2014;132(3):140 146. (doi: 10.1590/1516-3180.2014.1323568) 24760213 PMC10852089

[13] Laserna-Mendieta EJ Lucendo AJ. Faecal calprotectin in inflammatory bowel diseases: a review focused on meta-analyses and routine usage limitations. Clin Chem Lab Med. 2019;57(9):1295 1307. (doi: 10.1515/cclm-2018-1063) 30785706

[14] Shi JT Chen N Xu J Diagnostic accuracy of fecal calprotectin for predicting relapse in inflammatory bowel disease: a meta-analysis. J Clin Med. 2023;12(3):1206. (doi: 10.3390/jcm12031206) PMC9917450 36769850

[15] Nemeth E Ganz T. Hepcidin–ferroportin interaction controls systemic iron homeostasis. Int J Mol Sci. 2021;22(12):6493. (doi: 10.3390/ijms22126493) PMC8235187 34204327

[16] Ganz T. Hepcidin and iron regulation, 10 years later’. Blood. 2011;117(17):4425 4433. (doi: 10.1182/blood-2011-01-258467) 21346250 PMC3099567

